# 

**Published:** 1855-11

**Authors:** 


We again offer to our readers a number almost entirely original. The demands upon our space are such that we would willingly enlarge our size did other circumstances warrant it. The expenses of the Journal, however, are now much larger than heretofore. We have thus far made a very handsome and encouraging addition to our subscription list. But we want more. Will not our readers constitute themselves agents? We are constantly receiving letters assuring us that we are satisfying our patrons. If each of our present subscribers were to throw in his influence in our favor, and mention us kindly to his neighbor when he meets him in consultation, we should be better able, than now, to furnish a good Journal. We may be extravagant in our notions, but we believe that by spending a few hundred dollars more, per annum, on our Journal, we could make it the *best monthly in the country*.

---

Subscribers remitting money are requested to do so, hereafter, by "registered letter." Our losses by mail are so large that we are willing to try even this plan of prevention.

